# Development and pan-cancer validation of an epigenetics-based random survival forest model for prognosis prediction and drug response in OS

**DOI:** 10.3389/fphar.2025.1529525

**Published:** 2025-01-22

**Authors:** Chaoyi Yin, Kede Chi, Zhiqing Chen, Shabin Zhuang, Yongsheng Ye, Binshan Zhang, Cailiang Cai

**Affiliations:** ^1^ Department of Orthopaedics, Dongguan Hospital of Guangzhou University of Chinese Medicine, Dongguan, China; ^2^ Department One of Spine Surgery, Zhongshan Hospital of Traditional Chinese Medicine, Zhongshan, China

**Keywords:** osteosarcoma, epigenetic heterogeneity, single-cell RNA sequencing, random survival forest, prognostic model, drug sensitivity, pan-cancer analysis

## Abstract

**Background:**

Osteosarcoma (OS) exhibits significant epigenetic heterogeneity, yet its systematic characterization and clinical implications remain largely unexplored.

**Methods:**

We analyzed single-cell transcriptomes of five primary OS samples, identifying cell type-specific epigenetic features and their evolutionary trajectories. An epigenetics-based Random Survival Forest (RSF) model was constructed using 801 curated epigenetic factors and validated in multiple independent cohorts.

**Results:**

Our analysis revealed distinct epigenetic states in the OS microenvironment, with particular activity in OS cells and osteoclasts. The RSF model identified key predictive genes including OLFML2B, ACTB, and C1QB, and demonstrated broad applicability across multiple cancer types. Risk stratification analysis revealed distinct therapeutic response patterns, with low-risk groups showing enhanced sensitivity to traditional chemotherapy drugs while high-risk groups responded better to targeted therapies.

**Conclusion:**

Our epigenetics-based model demonstrates excellent prognostic accuracy (AUC>0.997 in internal validation, 0.832–0.929 in external cohorts) and provides a practical tool for treatment stratification. These findings establish a clinically applicable framework for personalized therapy selection in OS patients.

## 1 Introduction

Osteosarcoma (OS) is the most common primary malignant bone tumor, accounting for 56% of all bone sarcomas (Chen et al., 2021). The overall incidence rate is 4.5 per million (Siegel et al., 2024), with the incidence rate in the 0–24 age group significantly higher at 8.2 per million, showing a clear trend toward younger populations (Nie and Peng, 2018; Cole et al., 2022). OS primarily occurs in the metaphysis of long bones in extremities, particularly the distal femur, proximal tibia, and proximal humerus, with potential metastasis to adjacent bone tissues or distant organs (primarily lungs) (Chen et al., 2021; Yu and Yao, 2024). The disease originates from malignant transformation of osteoblasts (Aran et al., 2021), characterized by high invasiveness, early metastatic tendency, poor prognosis, and high rates of disability and mortality. Currently, the standard treatment protocol includes neoadjuvant chemotherapy, surgical resection, and consolidation chemotherapy (Thanindratarn et al., 2019). Despite recent advances in surgical techniques and chemotherapy regimens, while the 5-year relative survival rate for localized disease reaches 70%, 30%–40% of patients develop pulmonary metastases and recurrence, with post-progression survival rates dramatically declining to 20%–30% (Shaikh et al., 2016), resulting in no significant improvement in overall survival rates over the past decades (Mirabello et al., 2009; Czarnecka et al., 2020; Mialou et al., 2005). Therefore, elucidating metastatic mechanisms and predicting metastatic timing remain key challenges.

Epigenetic regulation plays a crucial role in gene expression and cell fate determination (Recillas-Targa, 2022). Its dysregulation can lead to gene dysfunction and malignant cell transformation, representing a key characteristic of tumor development. In OS, widespread alterations in DNA methylation patterns and histone modifications are observed (Tang et al., 2008), potentially contributing to tumorigenesis by interfering with mesenchymal stem cell differentiation into osteoblasts. Studies have shown that changes in methylation levels of tumor-suppressor microRNAs and hypomethylation of IGF2 growth factor and its promoter are closely associated with OS development (Azevedo et al., 2019). Unlike irreversible genetic mutations, epigenetic alterations are reversible, offering new therapeutic opportunities. However, due to the relative rarity of OS, research on its epigenetic mechanisms remains limited, hindering the establishment of precise prognostic models and personalized treatment strategies.

With advances in single-cell RNA sequencing technology (Grün and van Oudenaarden, 2015), analyzing tumor epigenetic heterogeneity at the single-cell level has become feasible. This study systematically evaluated epigenetic characteristics across different cell types in OS using single-cell sequencing data, revealing intercellular epigenetic differences and interaction networks. By integrating multiple large-scale datasets, we employed machine learning approaches, particularly random survival forest models (Rigatti, 2017), to construct an epigenetic feature-based risk scoring system and prognostic prediction model. Furthermore, we explored the model’s pan-cancer applicability and identified potential therapeutic targets through drug sensitivity analysis, providing new perspectives for precision medicine. This research not only deepens our understanding of epigenetic regulatory mechanisms in OS but also provides novel tools for patient prognostic assessment and individualized treatment decisions, holding significant clinical translational value. Notably, by incorporating immune microenvironment analysis, we further revealed associations between epigenetic alterations and tumor immune responses, offering new insights for optimizing immunotherapy strategies.

## 2 Materials and methods

**FIGURE 1:**
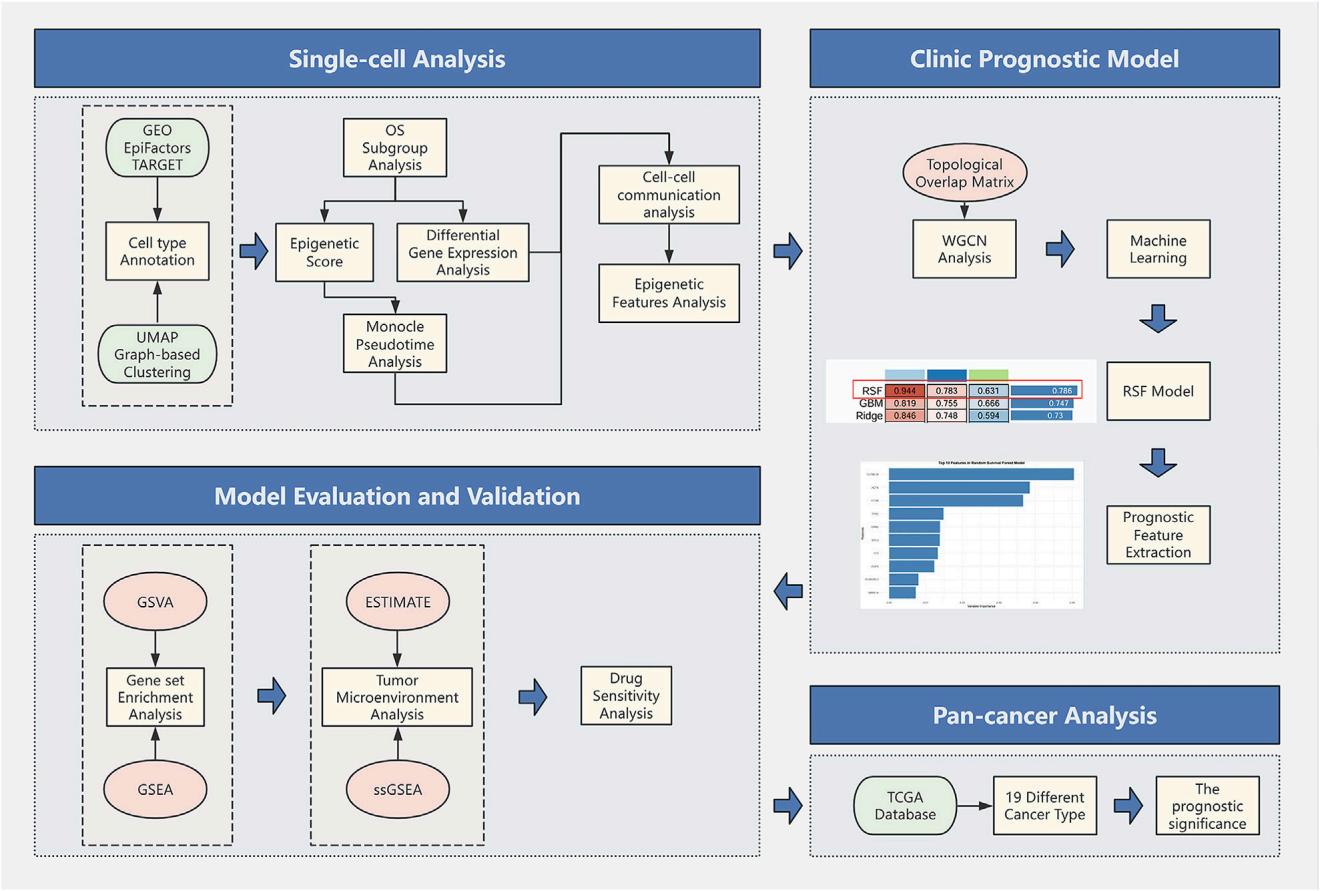
Study flowchart.

All analytical processes are illustrated in the flowchart (Figure 1).

### 2.1 Data source

In this study, we conducted single-cell RNA sequencing analysis on five primary OS samples (BC2, BC3, BC5, BC6, and BC16) obtained from the Gene Expression Omnibus database (GEO, https://www.ncbi.nlm.nih.gov/geo/, accession number: GSE152048). These samples were carefully selected from the original dataset containing 11 OS samples, specifically excluding recurrent and metastatic samples to minimize sample heterogeneity, following the clinical annotation provided by Zhou et al. (2021). To establish the epigenetic regulatory framework, we incorporated 801 epigenetics-related genes curated from the EpiFactors database (Medvedeva et al., 2015) (https://epifactors.autosome.org/). For validation purposes, we integrated additional OS datasets: expression profiles and survival information of 88 OS samples from the Therapeutically Applicable Research to Generate Effective Treatments (TARGET) Project (https://portal.gdc.cancer.gov/), and an independent cohort of 54 OS samples with survival data from GEO (accession number: GSE21257).

### 2.2 Single-cell analysis

#### 2.2.1 Data preprocessing and quality control

The single-cell RNA sequencing data analysis was performed using the Seurat package (version 5.0.0) in R (Huang et al., 2021). Initially, we filtered cells based on multiple quality control metrics: cells with less than 200 or more than 5,000 genes, UMI counts exceeding 20,000, mitochondrial gene percentage above 10%, and hemoglobin gene percentage above 1% were excluded. After quality control, gene expression matrices were normalized using the LogNormalize method, and the top 3,000 variable genes were identified for downstream analysis.

#### 2.2.2 Dimension reduction and batch effect correction

To minimize batch effects across different samples (Tran et al., 2020), we employed the Harmony algorithm for data integration (Korsunsky et al., 2019), followed by principal component analysis (PCA). The first 30 principal components were selected for uniform manifold approximation and projection (UMAP) dimensionality reduction and graph-based clustering (Becht et al., 2018) (resolution = 0.5).

#### 2.2.3 Cell type annotation and classification

Cell type annotation was performed using multiple approaches. We first utilized the SingleR package with the Human Primary Cell Atlas (https://www.humancellatlas.org/) as the reference database for automated annotation. This was followed by manual verification through examination of canonical cell type-specific markers: COL1A1, CDH11, and RUNX2 for OS cells; CTSK and MMP9 for osteoclastic cells; IL7R, CD3D, and NKG7 for tumor-infiltrating lymphocytes; CD74, CD14, and FCGR3A for myeloid cells; and PECAM1 and VWF for endothelial cells. Based on these analyses, we identified five major cell types in the tumor microenvironment: OS cells, osteoclastic cells, myeloid cells, endothelial cells, and tumor-infiltrating lymphocytes.

#### 2.2.4 Epigenetic score analysis and cell-cell communication

For epigenetic analysis, we calculated an epigenetic score for each cell using the single-sample Gene Set Enrichment Analysis (ssGSEA) method based on epigenetics-related genes obtained from the EpiFactors database Cells were then categorized into high and low epigenetic score groups based on the median score. Differential gene expression analysis between these groups was performed using the Wilcoxon rank-sum test, with adjusted p-value <0.05 and |log2FoldChange| > 0.5 as thresholds for significance. Cell-cell communication analysis was conducted using the CellChat package (Song et al., 2019), focusing on ligand-receptor interactions and signaling pathways between different cell types in both high and low epigenetic score groups (Jin et al., 2024).

#### 2.2.5 Trajectory analysis of OS cells

To investigate the developmental trajectory and potential state transitions of OS cells, we performed pseudotime analysis using Monocle2 (Hou et al., 2023). First, we subset the OS cells from the total cell population based on the previous cell type annotation. The expression matrix was converted to a Monocle object and filtered to retain genes expressed in at least 10 cells. Differentially expressed genes between high and low epigenetic score groups (adjusted p-value <0.05) were used as ordering genes for trajectory reconstruction. Dimensional reduction was performed using the DDRTree algorithm with default parameters, and cells were ordered along the trajectory. The root state was automatically determined based on the expression patterns of known early developmental markers. The resultant trajectory revealed distinct cell states and potential developmental paths of OS cells, which were visualized and colored by pseudotime, cell states, and cell subtypes to interpret the biological progression and heterogeneity of tumor cells.

### 2.3 Weighted gene co-expression network analysis

To explore the relationship between epigenetic scores and gene expression patterns, we performed Weighted Gene Co-expression Network Analysis (WGCNA) using the WGCNA R package (Liu et al., 2017; Langfelder and Horvath, 2008). The analysis was conducted on differentially expressed genes identified between high and low epigenetic score groups from the TCGA OS dataset. Prior to network construction, we filtered out genes with zero variance and samples with excessive missing values. The soft-thresholding power was determined by analyzing the scale-free topology fit index, with a power of five selected to achieve approximate scale-free topology (*R* ^2^ > 0.85). Network construction was performed using unsigned Topological Overlap Matrix (TOM) with a minimum module size of 50 genes and a merge cut height of 0.15 (Shuai et al., 2021). Module-trait relationships were assessed by correlating module eigengenes with epigenetic scores, and the significance of these correlations was determined using Student’s t-test. The pink module showed the strongest correlation with epigenetic scores (correlation coefficient = 0.75, p < 0.001). Gene significance and module membership were calculated to identify hub genes within the pink module, and the relationship between module membership and gene significance for epigenetics was visualized through scatter plots. The identified hub genes from the pink module were subsequently subjected to functional enrichment analysis to understand their biological implications in OS development.

### 2.4 Machine learning model development and validation

To establish a robust prognostic model, we employed multiple machine learning algorithms and their combinations. The expression data and survival information from TARGET database were randomly split into training (70%) and internal testing sets (30%). The GSE21257 dataset served as an independent external validation cohort. Prior to model development, all expression data were standardized using z-score normalization.

#### 2.4.1 Base models

• Random Survival Forest (RSF) with 1000 trees and node size of five
• Elastic Net (Enet) with nine different α values (0.1–0.9)
• Ridge regression (α = 0)
• Lasso regression (α = 1)
• CoxBoost regression
• Gradient Boosting Machine (GBM)
• Supervised Principal Components (SuperPC)
• Support Vector Machine for survival analysis (survival-SVM)

#### 2.4.2 Ensemble methods

We developed a series of RSF-based combination models and a hybrid Lasso-StepCox model to enhance prediction accuracy. The RSF was integrated with various statistical and machine learning approaches. For the CoxBoost combination, we optimized the penalty parameter through optimCoxBoostPenalty and determined optimal boosting steps via 10-fold cross-validation, ultimately employing an optimized penalty of 500. The Elastic Net series involved systematic variation of α values (0.1–0.9) with lambda optimization through cv.glmnet, selecting the best model based on minimal cross-validation error using 10-fold validation. For the GBM integration, we initialized 10,000 trees with interaction depth 3, minimum 10 observations per node, and 0.001 learning rate, optimizing the final tree number through cross-validation error.

The Lasso (α = 1) and Ridge (α = 0) combinations underwent 10-fold cross-validation with lambda optimization via cv.glmnet, utilizing RSF-selected features. The Stepwise Cox integration employed forward, backward, and bidirectional approaches with AIC-based selection criteria until reaching optimal model fit. For the SuperPC combination, we implemented feature standardization with 50th percentile threshold, developing a three-component model validated through 10-fold cross-validation, with threshold optimization based on cross-validated scores and single-component final prediction. The plsRcox integration determined optimal component numbers through 10-fold cross-validation, with selection based on prediction error minimization.

Additionally, we constructed a two-stage hybrid Lasso-StepCox model. The initial stage employed Lasso feature selection through elastic net regularization (α = 1), where predictive variables were transformed into matrix format alongside a survival outcome matrix. This Lasso implementation utilized 10-fold cross-validation via cv.glmnet under Cox proportional hazards specification, with optimal λ selection based on minimum cross-validation error. The second stage applied stepwise Cox regression to the Lasso-selected features in three directional approaches: forward selection (initiating with an empty model), backward selection (starting with all Lasso-selected variables), and bidirectional selection (combining both approaches). This hybrid approach leveraged both Lasso’s regularization and stepwise selection’s interpretability advantages.

#### 2.4.3 Model evaluation and validation strategy

Model performance was assessed using Harrell’s concordance index (C-index) (Cheung et al., 2019). For the training set (70% of TARGET data), we employed 10-fold cross-validation to avoid overfitting. Model performance was then evaluated on both the internal testing set (remaining 30% of TARGET data) and the external validation cohort (GSE21257). This multi-level validation strategy enables assessment of both internal generalizability within the TARGET cohort and external generalizability to independent datasets. Higher C-index values indicate better predictive accuracy, with one representing perfect prediction and 0.5 indicating random prediction.

#### 2.4.4 Optimal model analysis and validation

Among all tested algorithms, the Random Survival Forest (RSF) model demonstrated superior performance and was selected for detailed analysis. The final RSF model was constructed using the ‘randomForestSRC’ package with optimized parameters including 1000 trees and a node size of 5, employing log-rank splitting criteria to maximize survival differences between nodes. Variable selection was performed using a conservative ‘high’ threshold in the var.select function, which helped identify the most robust predictive features. The model utilized proximity matrices to assess sample similarity and out-of-bag (OOB) error estimates for internal validation.

Feature importance analysis was conducted using the built-in RSF algorithm’s Variable Importance (VIMP) scores, identifying and ranking the top 10 prognostic features based on their contribution to prediction accuracy. For risk stratification (Wang et al., 2023), we calculated individual risk scores using the RSF model’s mortality predictions and classified patients into high- and low-risk groups based on the median score. The model’s discriminative ability was assessed using time-dependent ROC curves at 1-, 3-, and 5-year time points, with AUC values calculated for both internal (30% TARGET) and external (GSE21257) validation cohorts. All statistical analyses were performed using R (version 4.1.0), with p < 0.05 considered statistically significant.

### 2.5 Pathway analysis and functional annotation

To investigate the biological implications of the RSF-based risk stratification, we performed comprehensive pathway analyses using multiple approaches. Gene set enrichment analysis (GSEA) was conducted using the Hallmark gene sets from MSigDB (Hanahan, 2022; Liberzon et al., 2015) (v2023.1). Differential expression analysis between high- and low-risk groups was performed using limma, with adjusted p-value <0.05 considered significant. Gene Set Variation Analysis (GSVA) was then applied to quantify pathway activities in individual samples. Pathway-specific survival analyses were performed using Kaplan-Meier estimates and log-rank tests to identify clinically relevant pathways. For significant pathways (log-rank p < 0.05), hazard ratios and 95% confidence intervals were calculated using Cox proportional hazards models. Correlation analysis was performed to explore the relationships between risk scores and pathway activities. All analyses were conducted using R with the clusterProfiler, GSVA, and survival packages.

### 2.6 Tumor microenvironment analysis

The tumor microenvironment (TME) characteristics between high- and low-risk groups were systematically evaluated using multiple approaches (Bejarano et al., 2021). We first employed the ESTIMATE algorithm to quantify stromal and immune cell infiltration levels, generating StromalScore, ImmuneScore, and ESTIMATEScore for each sample (Luo et al., 2020). The differences in these scores between risk groups were assessed using Wilcoxon rank-sum test.

For a more comprehensive understanding of immune-related pathways, we conducted ssGSEA (single-sample Gene Set Enrichment Analysis) using curated immune-related gene sets. The pathway activity differences between risk groups were evaluated using Student’s t-test, with p < 0.05 considered statistically significant. The results were visualized using heatmaps with row-wise z-score normalization.

Furthermore, for immune cell composition analysis, we employed a dual-method validation approach using the IOBR package (Zeng et al., 2021). Initially, immune cell abundance was quantified using ssGSEA based on a well-established set of 28 immune cell signatures. Subsequently, we validated these findings using the xCell algorithm, which provides an independent assessment of cellular composition in the tumor microenvironment. Cell-type-specific enrichment scores were calculated for both methods, and differences between risk groups were assessed using the Wilcoxon rank-sum test with Benjamini–Hochberg correction for multiple testing. Concordance between the two deconvolution methods was evaluated using Spearman’s correlation analysis. Only cell populations consistently identified as differentially abundant by both methods (adjusted P < 0.05) were considered robust findings. This dual-algorithm strategy was implemented to minimize method-specific biases and enhance the reliability of our immune cell infiltration analysis.

### 2.7 Drug sensitivity analysis

To explore potential therapeutic strategies for different risk groups, we performed drug sensitivity analysis using the pRRophetic package. Drug response predictions were based on the Genomics of Drug Sensitivity in Cancer (GDSC) database (2016 version) (https://www.cancerrxgene.org/) (Yang et al., 2013). The analysis pipeline was as follows: for each compound in the GDSC database, we predicted the half-maximal inhibitory concentration (IC50) values for each sample using ridge regression models trained on cancer cell line expression data (Sebaugh, 2011). The prediction model was selected through 10-fold cross-validation. Drug sensitivity differences between high- and low-risk groups were assessed using the Wilcoxon rank-sum test. Compounds showing significant differences (p < 0.05) in predicted IC50 values between risk groups were identified as potential therapeutic candidates. The results were visualized using boxplots with individual data points, and median differences in IC50 values between groups were calculated to indicate the direction and magnitude of sensitivity differences.

### 2.8 Pan-cancer analysis

To evaluate the broader applicability of our RSF-based prognostic model, we performed a comprehensive pan-cancer analysis across 19 different cancer types from The Cancer Genome Atlas (TCGA) database. Expression data and corresponding clinical information were obtained through the UCSC Xena platform. For each cancer type, we applied the following analysis pipeline: gene expression matrices were standardized and aligned with our model’s feature set. Missing genes were imputed with zero values after standardization. Risk scores were calculated using our established RSF model, and patients were stratified into high- and low-risk groups based on the median risk score. The prognostic significance was assessed using Cox proportional hazards regression and Kaplan-Meier survival analysis. Hazard ratios with 95% confidence intervals were calculated for each cancer type, and statistical significance was determined using the log-rank test (p < 0.05). Results were visualized using forest plots, incorporating hazard ratios, confidence intervals, and sample sizes for each cancer type. Cancer types with insufficient samples (n < 30) were excluded from the analysis.

## 3 Results

To investigate the cellular heterogeneity of the OS microenvironment, we performed single-cell RNA sequencing on five OS patient samples, yielding a total of 57,246 high-quality single cells (Supplementary Figures S1, S2). Following stringent quality control measures, we obtained 18,830 cells from patient BC16, 7,396 cells from BC2, 5,805 cells from BC3, 8,054 cells from BC5, and 17,161 cells from BC6. The median number of unique molecular identifiers (UMIs) ranged from 1,154 to 6,832 per cell across samples, with BC2 showing the highest transcriptional complexity (median 2,137 genes per cell). Despite some variation in sequencing depth among samples, with BC2 exhibiting higher UMI counts (mean 7,861) compared to others, all samples demonstrated robust gene detection rates (ranging from 221 to 4,944 genes per cell) suitable for downstream analysis. This comprehensive single-cell atlas provided a solid foundation for exploring the cellular composition and molecular characteristics of the OS tumor microenvironment.

### 3.1 Single-cell transcriptome analysis reveals cellular heterogeneity in OS microenvironment

**FIGURE 2:**
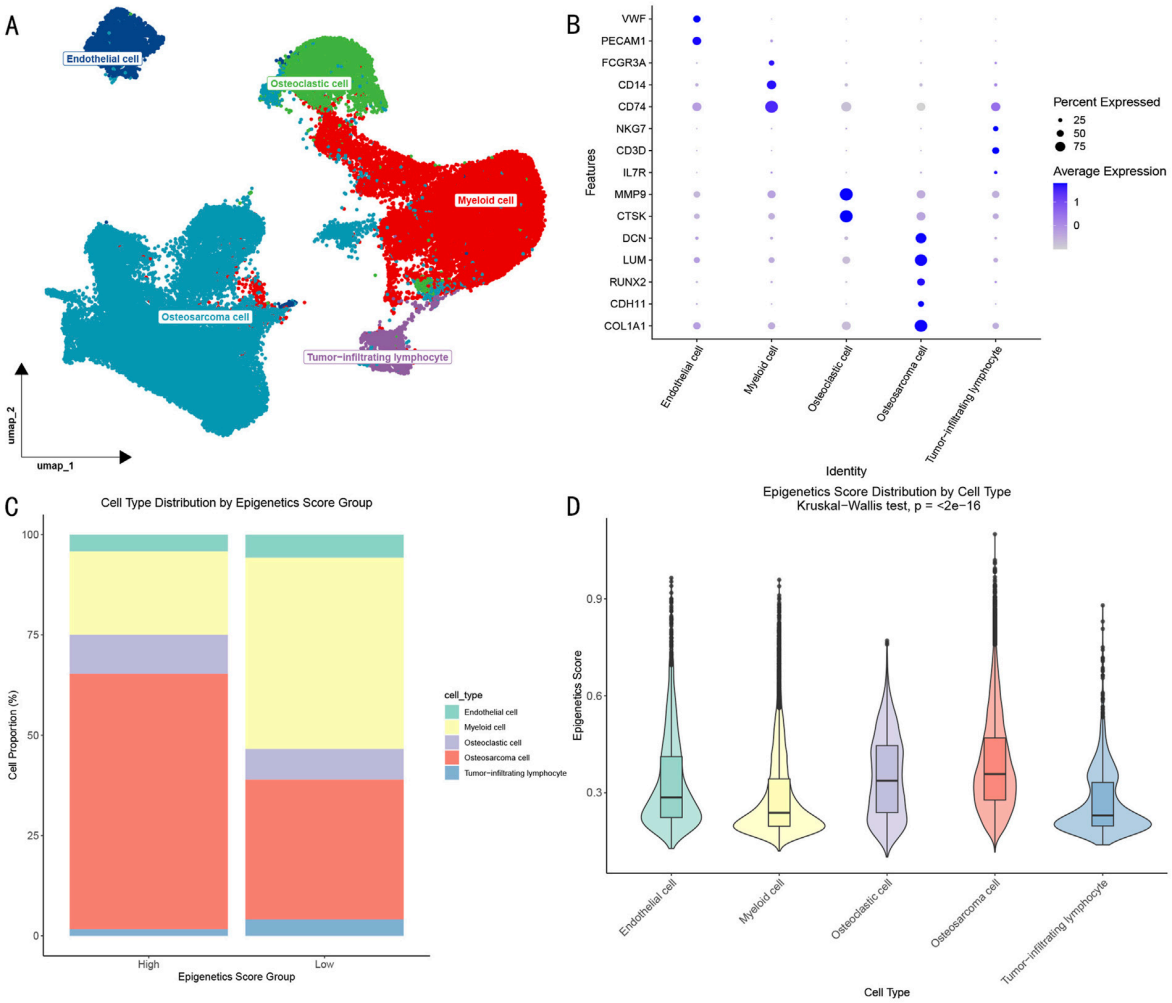
Epigenetic Characterization of OS Single-cell Transcriptome. **(A)** UMAP visualization showing the distribution of different cell subpopulations in the OS microenvironment. **(B)** Dotplot showing characteristic gene expression patterns of cell subpopulations. **(C)** Distribution proportions of cell subpopulations in high and low epigenetic score groups. **(D)** Differential analysis of epigenetic scores across cell subpopulations.

Through dimensional reduction clustering analysis and cell type annotation of single-cell RNA sequencing data (Figures 2A, B), we identified five major cell populations in the OS microenvironment: (1) OS cells expressing COL1A1, LUM, DCN, RUNX2, and CDH11; (2) myeloid cells with high expression of CD74, CD14, and FCGR3A; (3) osteoclasts specifically expressing MMP9 and CTSK; (4) endothelial cells enriched for PECAM1 and VWF expression; and (5) tumor-infiltrating lymphocytes expressing CD3D, NKG7, and IL7R. The identification of these cell subpopulations provided a foundation for understanding the cellular composition of the OS microenvironment.

Further analysis based on epigenetic scores divided cells into high- and low-score groups (Figure 2C). Although all 5 cell types were present in both groups, their proportions showed significant differences: the high-score group contained a significantly higher proportion of OS cells, while the low-score group showed enrichment of myeloid cells. Statistical analysis (Figure 2D) further revealed that OS cells and osteoclasts exhibited significantly higher epigenetic activity compared to other cell types (p < 0.05).

### 3.2 Pseudotime analysis reveals evolutionary trajectories of OS cells

**FIGURE 3:**
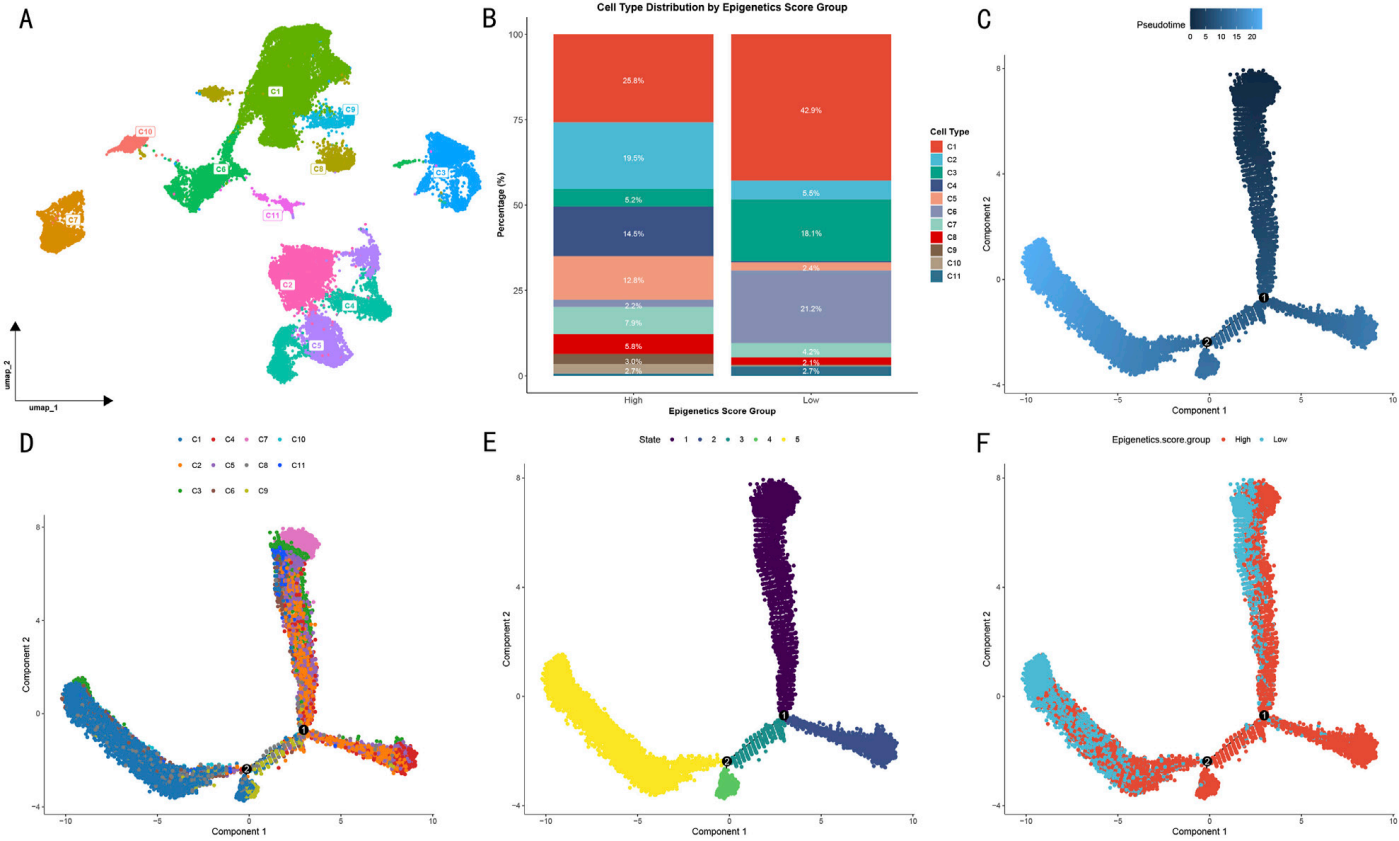
Pseudotime evolution analysis of OS cell subgroups. **(A)** UMAP visualization of cell subgroup distribution based on DDRTree algorithm. Different colors represent distinct transcriptional feature clusters. **(B)** Distribution proportions of 11 cell subgroups in high and low epigenetic score groups. **(C)** Cell pseudotime trajectory map. **(D)** Distribution of gene clusters along the evolutionary trajectory. **(E)** Cell evolutionary stage distribution. **(F)** Significant spatial separation pattern of high and low epigenetic score groups in evolutionary trajectory.

Through cell type differential analysis, we isolated the highest-scoring OS cells and identified 11 transcriptionally distinct gene clusters (Figure 3A). Based on epigenetic scores, samples were divided into high- and low-score groups (Figure 3B). In the high-score group, clusters C1 and C2 were predominant, accounting for 25.8% and 19.5% respectively, while in the low-score group, clusters C1 and C6 were most abundant, reaching 42.9% and 21.2% respectively.

To investigate the evolutionary trajectory of OS cells, we constructed a pseudotime landscape based on differentially expressed genes. Multi-dimensional visualization analysis revealed clear cell state transition patterns through pseudotime trajectory (Figure 3C), cell subtype distribution (Figure 3D), and evolutionary stages (Figure 3E). Specifically, among the 11 clusters, a unique evolutionary path emerged: initiating from clusters C2, C4, and C7, progressing through intermediate states, and ultimately transitioning to clusters C9 and C1. From a developmental perspective, cells progressed gradually from stages 1–2 through stage 3, ultimately reaching stages 4–5.

Notably, when cells were visualized according to their epigenetic scores (Figure 3F), high- and low-score groups showed significant spatial separation patterns in their evolutionary trajectories. This distribution pattern suggests that differences in epigenetic modification levels not only influence cellular phenotypes but also determine their positions and developmental directions in the evolutionary trajectory. The tendency of high- and low-score groups to occupy different evolutionary branches indicates that epigenetic regulation may be a key factor driving OS cell fate determination.

### 3.3 Cell-cell communication network analysis reveals functional characteristics of epigenetic regulation

**FIGURE 4:**
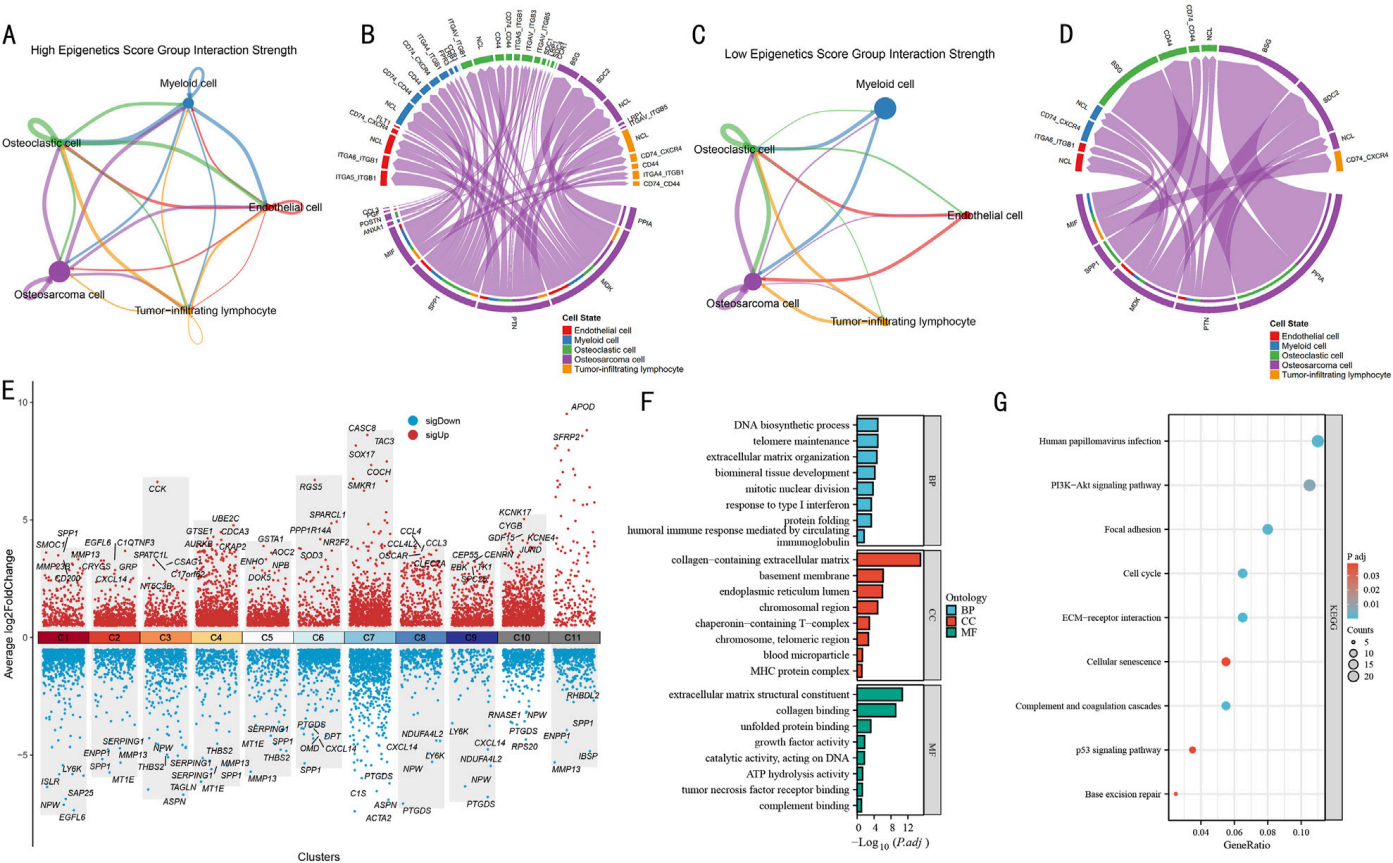
Cell-Cell communication network and functional pathway analysis in OS. **(A)** Cell signaling pathway network in the high epigenetic score group. Line thickness indicates interaction strength. **(B)** Circular plot of ligand-receptor interactions in the high-score group. Different colors represent different cell types, connecting lines indicate ligand-receptor pairs. **(C)** Cell signaling pathway network in the low epigenetic score group. Network structure shows significant simplification. **(D)** Circular plot of ligand-receptor interactions in the low-score group. Showing notably reduced interactions. **(E)** Volcano plot of differentially expressed genes across gene clusters. Red and blue indicate significantly up- and downregulated genes, respectively, with key differential genes labeled. **(F)** GO functional enrichment analysis results. Bar length represents enrichment significance (-log10P value). **(G)** KEGG pathway enrichment analysis results. Bubble size represents gene ratio, color intensity indicates significance (P value).

Using CellChat analysis, we systematically compared the differential features of cellular communication between high and low epigenetic score groups. In the high-score group, the 5 cell subpopulations exhibited complex interaction networks (Figure 4A). Notably, strong bidirectional communication was observed between OS cells and both osteoclasts and myeloid cells, while endothelial cells and tumor-infiltrating lymphocytes also demonstrated significant signal interactions. The corresponding ligand-receptor interaction map (Figure 4B) revealed activation of multiple key pathways, including immune regulatory pathways such as CD74-CD44 and ITGA4-ITGB1, as well as ligand-receptor pairs associated with extracellular matrix remodeling.

In contrast, the cell-cell communication network in the low-score group (Figure 4C) was significantly simplified, primarily showing limited interactions between OS cells and myeloid cells, with markedly reduced communication intensity among other cell types. The ligand-receptor interaction map (Figure 4D) also displayed a sparser molecular communication pattern, suggesting that reduced epigenetic modification levels may weaken intercellular signaling within the tumor microenvironment.

To decipher the molecular mechanisms of epigenetic regulation, we systematically analyzed the most significantly differentially expressed genes in each gene cluster (Figure 4E). Among all gene clusters, C1-C10 each displayed unique expression patterns. Particularly in the C1 cluster, which represented the terminal state in pseudotime analysis, we observed significant gene expression characteristics, including upregulation of SPP1, SMOC1, and MMP23B, and downregulation of LY6K, ISLR, and NPW.

GO functional enrichment analysis of the C1 cluster (Figure 4F) revealed multiple significantly enriched biological functions. In biological processes (BP), DNA biosynthetic process, telomere maintenance, and extracellular matrix organization were prominent, while humoral immune response and response to type I interferon suggested the importance of immune regulation. In cellular components (CC), collagen-containing extracellular matrix was most significant, along with enrichment of endoplasmic reticulum lumen and chromosomal region. In molecular functions (MF), extracellular matrix-related functions, represented by extracellular matrix structural constituent and collagen binding, were most prominent. KEGG pathway analysis (Figure 4G) further identified key signaling pathways, including PI3K-Akt signaling pathway, Focal adhesion, p53 signaling pathway, Cell cycle, and Base excision repair.

### 3.4 WGCNA analysis identifies key epigenetic regulatory modules

**FIGURE 5:**
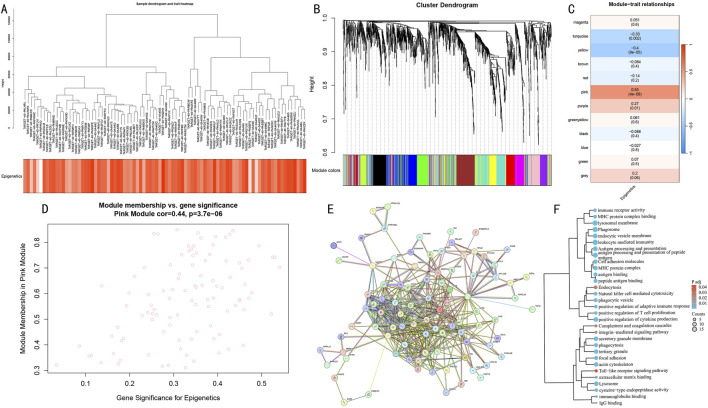
WGCNA network analysis reveals key gene modules of epigenetic modification. **(A)** Hierarchical clustering dendrogram of differential genes and heatmap of epigenetic score distribution. **(B)** Identification results of 12 functional modules. **(C)** Module-trait correlation analysis. Pink module shows strongest positive correlation. **(D)** GS-MM correlation analysis of the Pink module. **(E)** PPI network of differential genes. **(F)** Functional enrichment clustering analysis of core genes in the Pink module.

To systematically identify co-expression networks associated with epigenetic modifications, we performed WGCNA analysis on differentially expressed genes. Hierarchical clustering results demonstrated gene co-expression relationships and sample epigenetic score distribution (Figure 5A). Using the dynamic tree-cutting algorithm, we identified 12 functional modules (Figure 5B). Module-trait correlation analysis revealed that the Pink module showed the strongest positive correlation with epigenetic scores (cor = 0.55, P = 4e-8) (Figure 5C), and genes within this module demonstrated significant correlation between gene significance (GS) and module membership (MM) (cor = 0.44, P = 3.7e-06) (Figure 5D).

Further protein-protein interaction network construction (Figure 5E) and functional enrichment analysis (Figure 5F) revealed that core genes in the Pink module were primarily enriched in immune response-related pathways, including antigen processing and presentation, leukocyte mediated immunity, and natural killer cell mediated cytotoxicity; cellular vesicle transport processes such as endocytosis and lysosome pathway; and cell adhesion-related functions including cell adhesion molecules and focal adhesion. These results suggest that the Pink module may serve as a core functional module coordinating the epigenetic regulatory network.

### 3.5 Development of random survival forest-based prognostic prediction model

**FIGURE 6:**
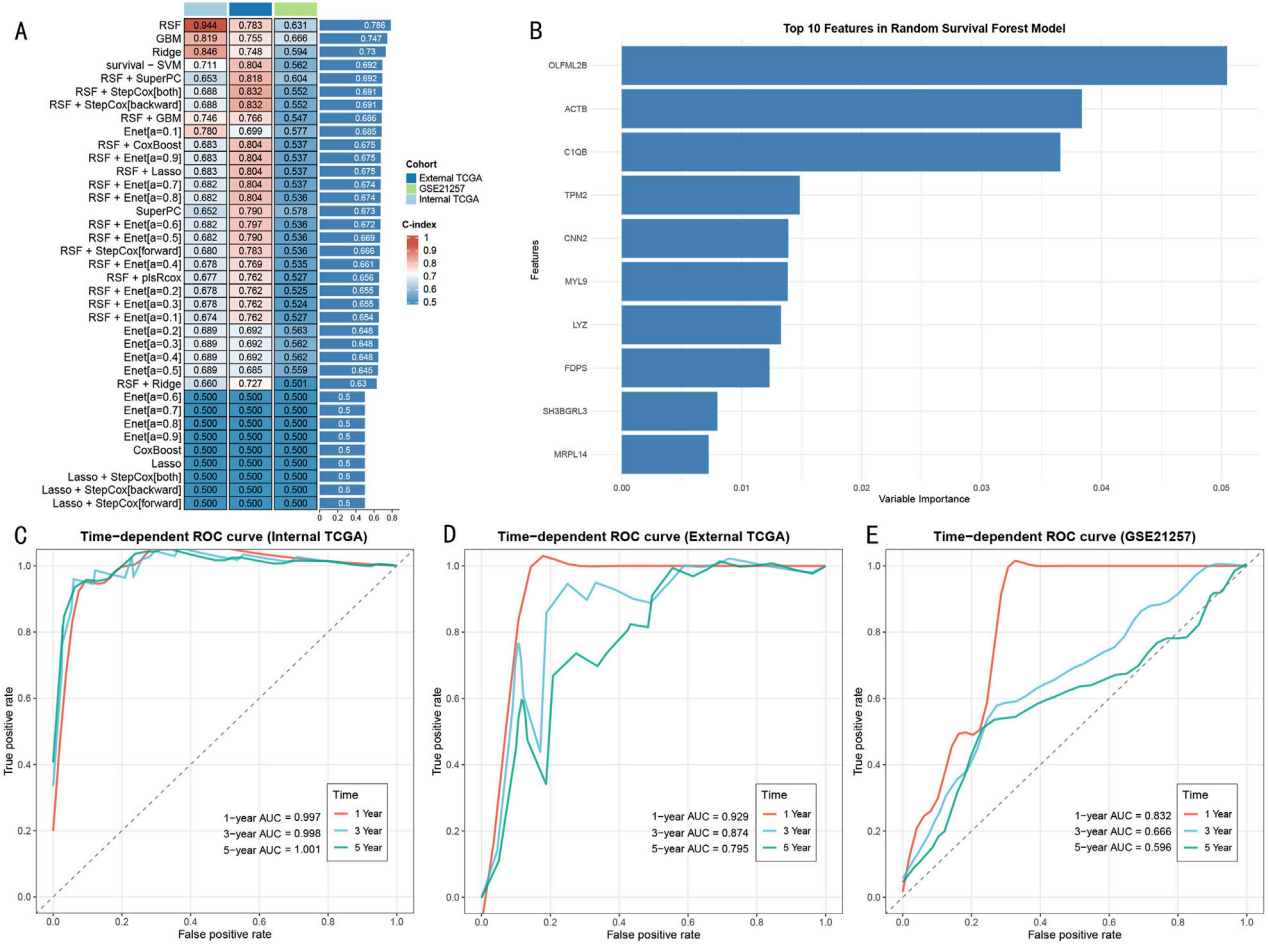
Construction and Validation of RSF Prognostic Prediction Model **(A)** Performance evaluation heatmap of 37 machine learning algorithms. Color intensity indicates C-index value, with RSF model showing optimal performance across three cohorts. **(B)** Importance score ranking of top 10 feature genes in RSF model, with OLFML2B, ACTB, and C1QB being the three most contributive genes. **(C)** Time-dependent ROC curves in TCGA training set, showing extremely high prediction accuracy (AUC>0.997). **(D)** ROC curves in external TCGA validation set, maintaining good predictive performance (AUC: 0.795–0.929). **(E)** ROC curves in GSE21257 validation set, confirming external applicability of the model (AUC: 0.596–0.832).

Through systematic evaluation of eight machine learning algorithms and their various combinations (Figure 6A), the RSF model demonstrated superior predictive performance across three cohorts (internal TCGA: 0.944, external TCGA: 0.783, GSE21257: 0.631). Feature importance analysis identified 10 core predictive genes (Figure 6B), with OLFML2B, ACTB, and C1QB showing the highest variable importance scores. Model performance evaluation revealed significant time-dependent predictive capability: The RSF model demonstrated excellent discriminative ability in the TCGA internal training set, with AUC values of 0.997, 0.998, and 1.001 for 1-year, 3-year, and 5-year survival predictions, respectively (Figure 6C). This predictive performance was validated in external validation cohorts, with AUC values in the external TCGA cohort of 0.929 (1-year), 0.874 (3-year), and 0.795 (5-year) (Figure 6D), and in the GSE21257 cohort of 0.832 (1-year), 0.666 (3-year), and 0.596 (5-year) (Figure 6E), demonstrating stable predictive efficiency and promising clinical application potential.

Notably, compared to other machine learning methods (such as GBM: 0.819, Ridge: 0.846, survival-SVM: 0.711), the RSF model showed significant advantages in the internal validation set. This predictive advantage was maintained in external validation cohorts, confirming the model’s robustness and generalizability.

### 3.6 Functional mechanism analysis of RSF risk stratification

**FIGURE 7:**
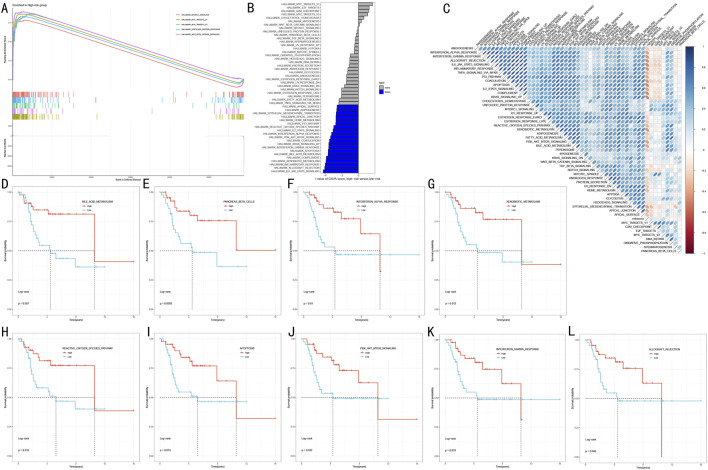
Functional Mechanism Analysis of RSF Risk Stratification **(A)** GSEA analysis showing key pathway enrichment patterns in high-risk group **(B)** Waterfall plot of differential pathways revealed by GSVA analysis **(C)** Correlation heatmap between risk scores and pathway activities **(D–L)** Kaplan-Meier survival analysis curves for 9 key pathways.

To elucidate the molecular biological basis of RSF risk stratification, we employed a multi-level functional enrichment analysis strategy. GSEA revealed that the high-risk group was significantly enriched in multiple cancer-related Hallmark pathways (Figure 7A), including Mtorc1 signaling, MYC targets V1/V2, Unfolded protein response, and Wnt beta catenin signaling (FDR <0.05). GSVA further revealed distinct pathway characteristics of risk stratification (Figure 7B). Notably, multiple immune-related pathways were significantly downregulated in the high-risk group, including IL6-JAK-STAT3 signaling (t = −4.21, adj.P.Val = 0.002), inflammatory response (t = −3.97, adj.P.Val = 0.002), and interferon responses (gamma: t = −3.55, adj.P.Val = 0.004; alpha: t = −3.22, adj.P.Val = 0.008). Additionally, several critical cancer-associated pathways showed significant depletion in the high-risk group, including allograft rejection (t = −4.03, adj.P.Val = 0.002), complement (t = −3.84, adj.P.Val = 0.002), and PI3K-AKT-MTOR signaling (t = −3.34, adj.P.Val = 0.006). Correlation analysis between risk scores and pathway activities (Figure 7C) validated these findings.

To assess the clinical prognostic significance of key pathways, we selected nine most significant signaling pathways for survival analysis (Figures 7D–L). Kaplan-Meier analysis showed that high activity in these pathways was significantly associated with better prognosis (P < 0.05):

• Metabolism-related pathways: Bile acid metabolism (p = 0.037) and Xenobiotic metabolism (p = 0.012).
• Immune-related pathways: Interferon alpha/gamma response (p = 0.031/p = 0.033) and Allograft rejection (p = 0.042).
• Cell death pathways: Reactive oxygen species pathway (p = 0.016) and Apoptosis (p = 0.016).
• Signal transduction pathways: PI3K-AKT-MTOR signaling (p = 0.029) and Pancreas beta cells (p = 0.008).

### 3.7 Tumor microenvironment characteristic analysis

**FIGURE 8:**
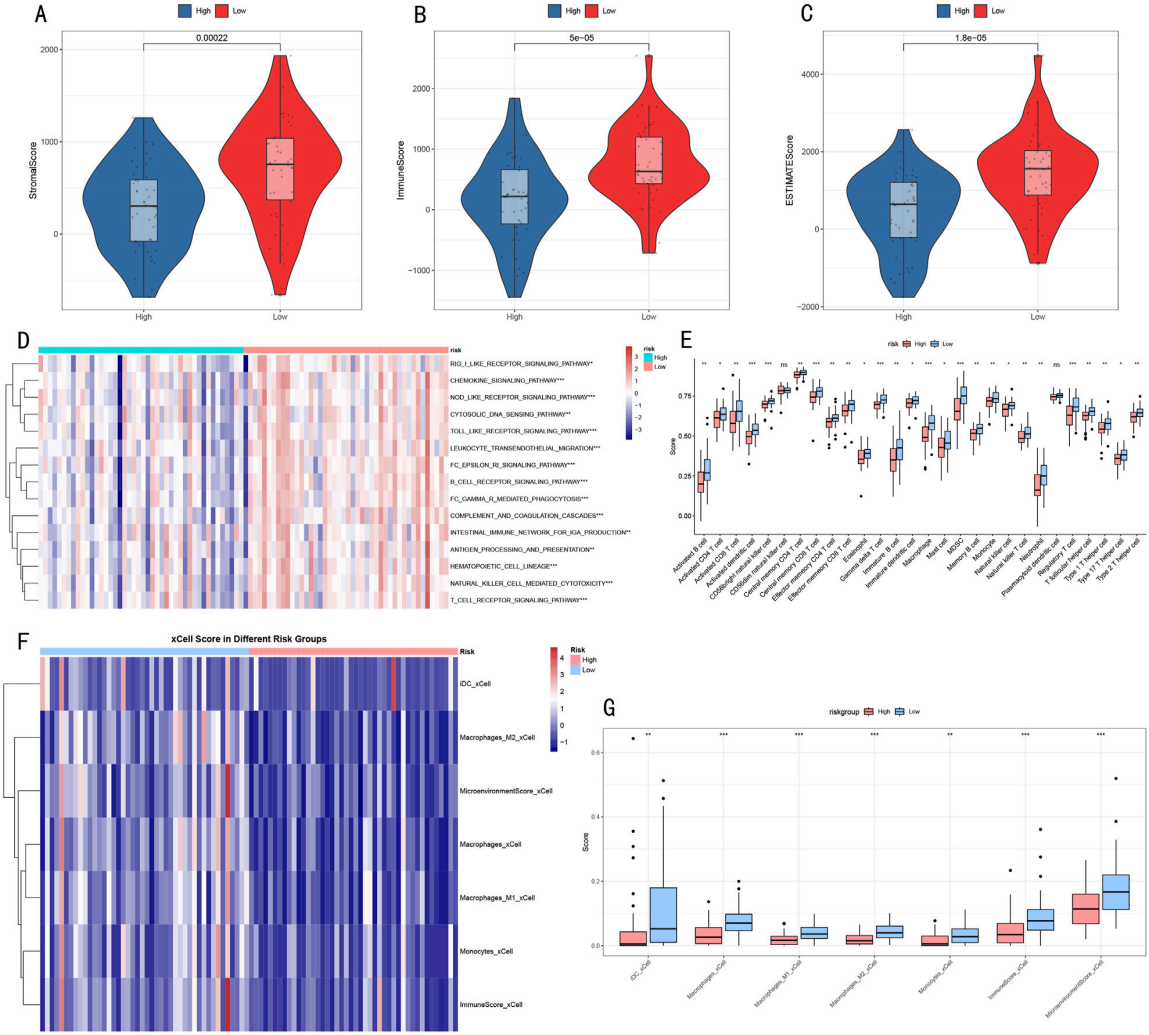
Multi-dimensional Analysis of Tumor Microenvironment Characteristics **(A–C)** Violin plots showing microenvironmental component differences in ESTIMATE scores **(D)** Heatmap of immune-related pathway activities **(E)** Box plots of immune cell infiltration levels. **(F)** Heatmap showing significantly different immune cell populations identified by xCell analysis between risk groups. **(G)** Box plots depicting the abundance of significant immune cell populations from xCell analysis. *p < 0.05, **p < 0.01, ***p < 0.001.

To better understand the relationship between RSF risk stratification and tumor microenvironment, we employed a multi-dimensional analysis strategy to evaluate microenvironmental differences between high- and low-risk groups. ESTIMATE algorithm analysis showed that compared to the high-risk group, the low-risk group had significantly elevated microenvironment scores: StromalScore (p = 0.00022, Figure 8A), ImmuneScore (p = 5e-05, Figure 8B), and ESTIMATEScore (p = 1.8e-05, Figure 8C) all showed significant differences.

Immune-related pathway analysis (Figure 8D) revealed 15 signaling pathways with significant differences between high- and low-risk groups, primarily including:

• Receptor signaling pathways: BCR/TCR receptor signaling pathway
• Cell migration-related: Leukocyte transendothelial migration
• Immune response processes: Complement and coagulation cascade, Natural killer cell mediated cytotoxicity
• Signal transduction: Toll-like receptor signaling pathway, Cytokine signaling pathway

Further immune cell infiltration analysis using complementary approaches revealed consistent patterns in the tumor microenvironment. Initial analysis of 28 immune cell subgroups (Figure 8E) showed distinct abundance differences between high- and low-risk groups. These findings were further validated by xCell analysis (Figures 8F, G), which demonstrated significantly decreased infiltration levels of multiple myeloid cell populations in the high-risk group, including dendritic cells (iDC), macrophages (both M1 and M2 subtypes), and monocytes (all P < 0.05). Notably, the comprehensive microenvironment and immune scores from xCell analysis also confirmed lower immune cell infiltration in the high-risk group, consistent with our ESTIMATE and 28 immune cell subgroups analysis findings. This convergence of results from multiple analytical approaches suggests a systematic difference in immune cell composition between risk groups, characterized by reduced myeloid cell infiltration in high-risk tumors.

### 3.8 Drug sensitivity analysis based on risk stratification

**FIGURE 9:**
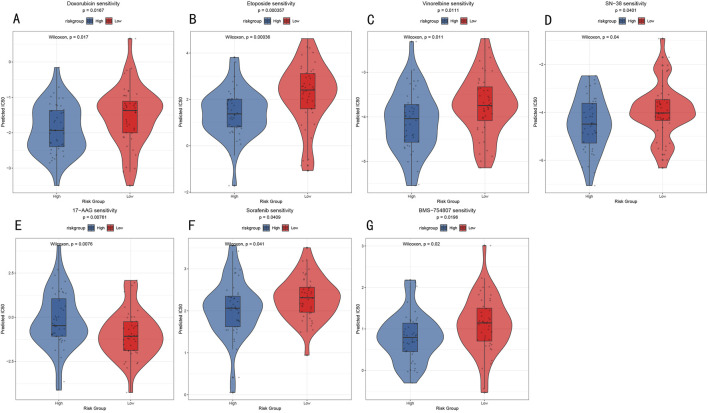
Drug Sensitivity Analysis **(A–G)** Violin plots showing IC50 value distribution of seven key drugs between high- and low-risk groups. *Y*-axis represents predicted IC50 values, with lower IC50 values indicating higher drug sensitivity.

To explore the potential value of the RSF risk stratification model in guiding individualized treatment, we analyzed drug sensitivity differences between high- and low-risk groups. Through comparison of predicted IC50 values, we identified seven drugs showing significant sensitivity differences (Figure 9), including traditional chemotherapy drugs Doxorubicin (anthracycline, p = 0.0167, Figure 9A), Etoposide (topoisomerase inhibitor, p = 0.00036, Figure 9B), Vinorelbine (microtubule inhibitor, p = 0.011, Figure 9C), and SN-38 (topoisomerase I inhibitor, p = 0.04, Figure 9D), as well as targeted therapeutic agents 17-AAG (Hsp90 inhibitor, p = 0.0076, Figure 9E), Sorafenib (multi-target tyrosine kinase inhibitor, p = 0.041, Figure 9F), and BMS-754807 (IGF-1R/IR inhibitor, p = 0.02, Figure 9G). Notably, the low-risk group demonstrated higher sensitivity to most chemotherapy drugs, particularly showing the most significant responses to Etoposide and SN-38 (Figures 9B, D). These results provide a theoretical foundation for RSF risk stratification-based personalized medication strategies while revealing that tumors with different molecular characteristics may require differentiated treatment approaches.

### 3.9 Pan-cancer prognostic value analysis of the RSF model

**FIGURE 10:**
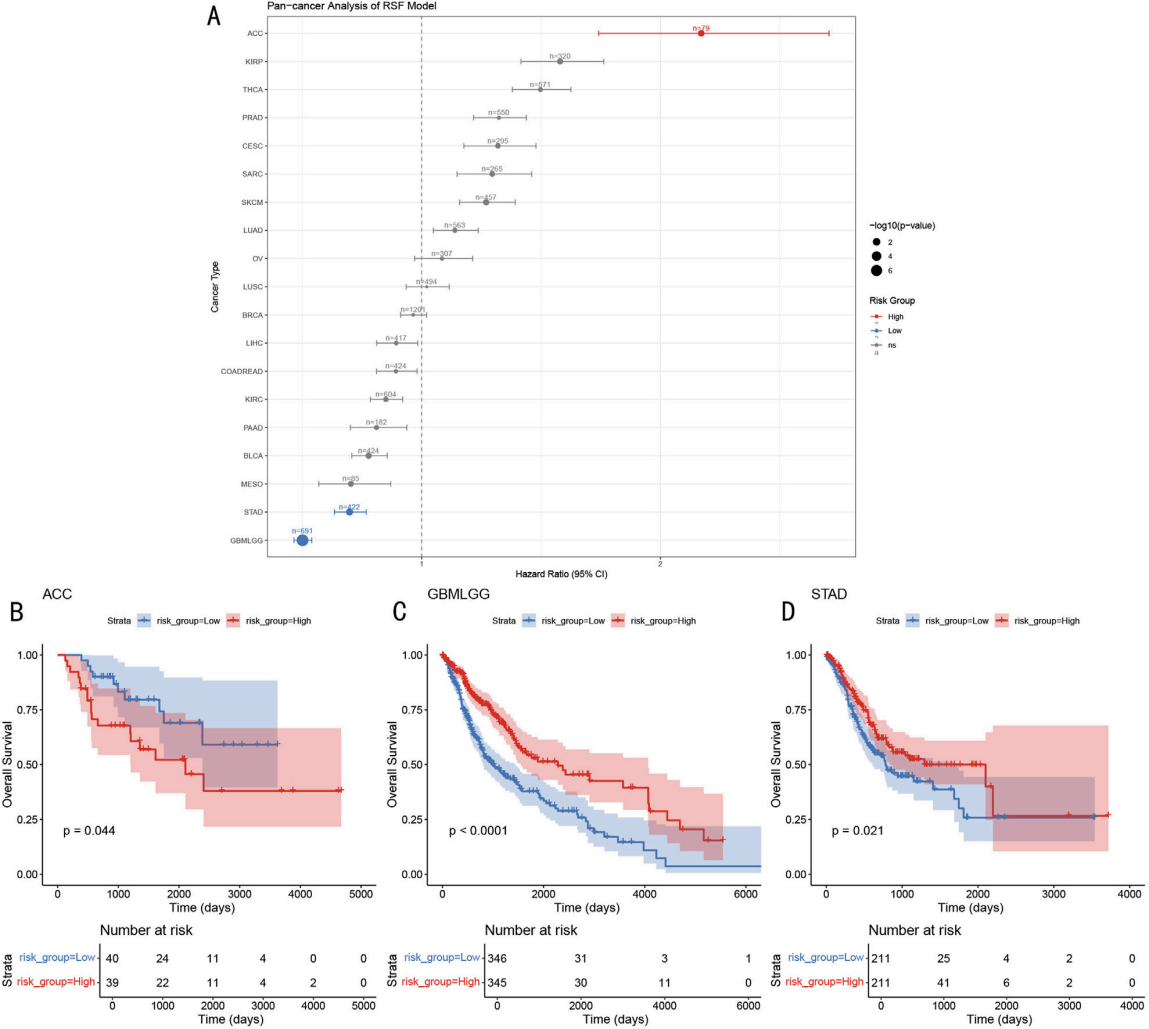
Pan-cancer Prognostic Prediction Analysis of RSF Model **(A)** Forest plot of hazard ratios for 19 cancer types, dot size represents -log10 (p-value). **(B–D)** Kaplan-Meier survival curves for ACC, GBMLGG, and STAD, including risk group stratification and temporal changes in patient numbers.

To evaluate the applicability of the RSF prognostic model in other cancer types, we conducted systematic analysis across 19 different cancer types from the TCGA database (Figure 10A). Results showed that the model demonstrated significant prognostic value in multiple cancer types, including ACC (Adrenocortical Carcinoma, HR > 2), GBMLGG (Glioma, HR < 1), and STAD (Gastric Adenocarcinoma, HR < 1). Further survival analysis revealed that in ACC, high-risk group patients had significantly shorter overall survival compared to the low-risk group (p = 0.044, Figure 10B), while GBMLGG and STAD showed opposite trends, with high-risk group patients showing better prognosis (GBMLGG: p < 0.0001, STAD: p = 0.021; Figures 10C, D). This differential prognostic pattern suggests that the RSF model may capture common molecular features across different cancer types, but their biological significance may vary in different tissue contexts.

## 4 Discussion

### 4.1 Epigenetic heterogeneity and tumor progression

Through single-cell epigenetic analysis, our study revealed the crucial role of epigenetic regulation during OS progression. Regarding cell fate determination, we observed that variations in epigenetic scores significantly influenced the distribution patterns across eleven transcriptional subgroups. The high-score group was predominantly characterized by C1 (25.8%) and C2 (19.5%) subgroups, while the low-score group was dominated by C1 (42.9%) and C6 (21.2%) subgroups. This distributional disparity suggests that epigenetic modifications may influence cell differentiation trajectories through the regulation of specific transcriptional programs. Notably, pseudotime analysis revealed that the transition of cells from early C2, C4, and C7 subgroups to late C9 and C1 subgroups is strictly regulated by epigenetic modification levels, indicating that epigenetic reprogramming acts as a “molecular switch” in tumor progression (Sun et al., 2022).

At the cellular communication network level, the high-score group exhibited more complex and active signaling interaction patterns. Particularly noteworthy was the intense bidirectional communication between OS cells and osteoclasts/myeloid cells, alongside the activation of key immunoregulatory pathways involving CD74-CD44 and ITGA4-ITGB1. CD74-CD44 signaling promoted immune cell recruitment and activation, while ITGA4-ITGB1 facilitated immune cell adhesion and migration within the tumor microenvironment. In contrast, the cellular communication network in the low-score group was notably simplified, primarily limited to restricted interactions between OS cells and myeloid cells. This likely represents more than mere correlation, suggesting that epigenetic modifications actively shape the immune microenvironment through the regulation of these pathways (Yang et al., 2023). This finding provides new perspectives for understanding tumor immune escape mechanisms (Rodríguez et al., 2003) while suggesting that epigenetic interventions targeting these pathways may hold potential for modulating the tumor immune microenvironment.

From an evolutionary perspective, epigenetic modifications demonstrated strong associations with tumor evolution. Our pseudotime analysis clearly illustrated spatial separation patterns of cells along evolutionary trajectories, with high-score and low-score groups tending to occupy distinct evolutionary branches. This phenomenon suggests that epigenetic modifications may drive tumor cells along specific evolutionary paths by influencing dynamic changes in gene expression profiles. Particularly in the terminal C1 subgroup, we observed significant upregulation of key genes including SPP1, SMOC1, and MMP23B. Functional enrichment analysis of these genes further revealed the involvement of crucial biological processes such as DNA replication, telomere maintenance, and extracellular matrix remodeling, potentially representing important mechanisms by which cells with high epigenetic activity maintain homeostasis (Budhavarapu et al., 2013; Hakobyan et al., 2024).

### 4.2 Innovation and clinical translation value of the prognostic model

Our developed RSF prognostic model demonstrates three distinctive advantages: First, compared to traditional Cox proportional hazards models and other machine learning methods, the RSF model automatically handles non-linear relationships and higher-order interactions, achieving outstanding predictive accuracy (C-index of 0.944). Second, the model integrates epigenetic regulatory features, considering not only gene expression levels but also dynamic changes in epigenetic modifications, providing a new dimension for capturing tumor heterogeneity. Third, the model demonstrates consistent predictive performance across multiple independent cohorts, particularly maintaining consistency in predictions at different time points (1-year, 3-year, and 5-year), a time-dependent predictive characteristic crucial for clinical decision-making.

In terms of personalized treatment, our study is the first to reveal the possibility of differentiated treatment strategies based on RSF risk stratification. Notably, we observed significant differences in drug sensitivity between high-risk and low-risk groups: the low-risk group showed higher sensitivity to conventional chemotherapy agents (such as DNA topoisomerase inhibitors Etoposide and SN-38), possibly related to their higher cell proliferation activity and DNA replication dependency. The enhanced sensitivity to topoisomerase inhibitors in the low-risk group may be attributed to their more active DNA replication machinery and higher expression of topoisomerase-related genes, making them more vulnerable to DNA damage-induced cell death. In contrast, the high-risk group demonstrated better responses to molecular targeted drugs (such as Hsp90 inhibitor 17-AAG and multi-kinase inhibitor Sorafenib), correlating with their activated specific signaling pathways. For instance, 17-AAG may influence the stability of multiple epigenetic regulatory factors through Hsp90 inhibition (Talaei et al., 2019), while Sorafenib, as a multi-target inhibitor, might affect epigenetic modification processes by interfering with RAF/MEK/ERK and PI3K/AKT signaling pathways (Abdelgalil et al., 2019; Ullah et al., 2022; Manning and Toker, 2017). The superior response to targeted therapies in the high-risk group could be explained by their greater dependence on these specific molecular pathways for survival and proliferation, as evidenced by their distinct pathway activation signatures and epigenetic profiles. This differential response pattern suggests that RSF risk scores may reflect fundamental biological characteristics and signaling pathway dependencies of tumor cells, which directly determine cellular sensitivity to drugs with different mechanisms of action.

However, clinical application of drug sensitivity prediction faces several key challenges: First, can *in vitro* prediction results accurately reflect *in vivo* drug responses, particularly considering factors such as drug metabolism and bioavailability? Second, given the complexity of the tumor microenvironment, is prediction based solely on epigenetic features sufficiently comprehensive? For example, our observed differences in immune microenvironment might affect the efficacy of immune checkpoint inhibitors, while the activation level of angiogenesis-related pathways might influence the effectiveness of anti-angiogenic drugs. Additionally, metabolic reprogramming and stress response mechanisms of tumor cells might affect treatment outcomes through their influence on drug transport and detoxification. These issues require validation through prospective clinical studies. Nevertheless, our model provides an actionable framework for developing personalized treatment plans, particularly valuable in selecting first-line treatment strategies and predicting therapeutic responses.

Notably, the core feature genes identified by the model (such as OLFML2B, ACTB, and C1QB) may serve not only as predictive markers but also as potential therapeutic targets. Their central position in the epigenetic regulatory network suggests that targeted interventions against these molecules might produce cascade effects, thereby affecting the activity of entire signaling networks. This finding provides direction for developing new therapeutic strategies, particularly in considering combination therapy regimens, where individualized adjustments can be made based on the expression patterns of these core genes.

### 4.3 Interaction between immune microenvironment and epigenetic regulation

This study reveals complex interactions between epigenetic features and the immune microenvironment through integrated analysis. We found that the low-risk group exhibited significantly elevated immune and stromal scores, a seemingly counterintuitive phenomenon that yields interesting insights upon deeper analysis: high epigenetic activity may promote tumor immune evasion by suppressing immune cell recruitment and activation. Specifically, GSEA analysis revealed significant activation of MYC and mTORC1 signaling pathways in the high-risk group, which are known to reshape the tumor immune microenvironment, suppress T cell function, and promote myeloid-derived suppressor cell accumulation (Dhanasekaran et al., 2022; Kim et al., 2017).

Regarding epigenetic modification’s regulation of immune responses, we observed several key mechanisms. Firstly, CD74-CD44 signaling pathway activation in the high epigenetic activity group suggests that epigenetic modifications may influence immune recognition through antigen presentation regulation. Then, differential expression of the ITGA4-ITGB1 pathway indicates potential epigenetic influence on immune cell chemotaxis and infiltration. Specifically, CD74-CD44 signaling may promote immune evasion through multiple mechanisms: CD74 can regulate MHC class II trafficking and antigen loading, while CD44 engagement can trigger immunosuppressive cytokine production and regulatory T cell expansion. The ITGA4-ITGB1 pathway activation could facilitate selective immune cell recruitment, favoring immunosuppressive cell populations like MDSCs and Tregs while impeding cytotoxic T cell infiltration. Besides, the immune cell communication network remodeling observed at the single-cell level may represent a novel immune evasion mechanism: cells with high epigenetic activity alter immune cell signaling to weaken the synergistic effects of anti-tumor immune responses.

These findings suggest new approaches for optimizing immunotherapy strategies:

1. Epigenetic-immune combination therapy (Liang et al., 2023): For high-risk patients, epigenetic modulator pretreatment followed by immunotherapy might achieve better therapeutic outcomes.
2. Personalized immunotherapy: Patient epigenetic characteristics could more accurately predict immune checkpoint inhibitor response, guiding optimal immunotherapy strategy selection.
3. Microenvironment remodeling: Targeting key epigenetic regulators might reshape the immunosuppressive microenvironment, enhancing immunotherapy efficacy.

However, integrating single-cell and TCGA database analyses revealed an apparently contradictory but enlightening phenomenon: at the single-cell level, the high epigenetic score group showed more active immune pathways and complex cell communication networks, while TCGA cohort analysis showed higher immune scores and immune cell infiltration in the low-risk group. This discrepancy may reflect scale-specific epigenetic regulation effects: single-cell analysis captures microscopic instantaneous states, while TCGA data reflects macroscopic average states. Further analysis reveals a consistent pattern of immune suppression in the high-risk group, characterized by significantly decreased infiltration of various immune cell populations and downregulation of immune-related pathways. This comprehensive immune deficiency, rather than active immune suppression, may create a “cold” tumor microenvironment that favors tumor progression. The systematic reduction in both innate (macrophages, dendritic cells) and adaptive immune components suggests an immune-desert phenotype in high-risk tumors, which could explain their poor prognosis and potentially guide immunotherapy strategies.

From a dynamic equilibrium perspective, high immune pathway activity observed at the single-cell level may represent immediate stress responses to immune pressure, while low immune infiltration at the tissue level may be the final effect of this stress response, suggesting that epigenetic modifications may maintain an immune microenvironment state favorable for tumor survival through dynamic regulation. This finding not only explains the scale-dependent characteristics of epigenetic regulation but also provides new perspectives for understanding tumor immune evasion mechanisms.

### 4.4 Mechanism discussion for pan-cancer application

The opposing prognostic significance of epigenetic modifications across different cancer types likely reflects the tissue specificity and microenvironment dependency of epigenetic regulation. In endocrine system tumors (such as ACC), high levels of epigenetic activity often correlate with dedifferentiation and invasive phenotypes (Ettaieb et al., 2020), possibly due to endocrine tissues’ high dependency on epigenetic balance. Endocrine cells require precise epigenetic regulation to maintain their specialized functions and hormone secretion capabilities; thus, epigenetic imbalance may directly lead to cellular dysfunction and malignant progression. In contrast, in nervous system tumors (GBMLGG) and digestive system tumors (STAD), high epigenetic activity may instead represent better differentiation states and tissue homeostasis maintenance capability (Li et al., 2019; Hong et al., 2021).

These differences may stem from the developmental origins and metabolic characteristics of these tissues: glial cells inherently possess higher plasticity, where moderate epigenetic activity may help maintain their normal function; gastric mucosal cells require continuous renewal and differentiation processes, where higher epigenetic activity may reflect better tissue homeostasis regulation.

## 5 Limitation and future perspectives

Although this study revealed significant characteristics of epigenetic heterogeneity in OS and established an effective prognostic prediction model, several limitations need to be addressed in future research. First, the current single-cell analysis faces both technical and sample size limitations. The technical constraints include potential cell dropout effects, limited capture of rare cell populations, and computational challenges in data processing. The small sample size may not fully capture the complete landscape of epigenetic heterogeneity in OS, necessitating expanded cohort sizes and integration of multi-omics data. Second, detailed patient clinical characteristics, including complete follow-up information and treatment protocols, were not available in the current database, which limits our ability to conduct comprehensive clinical correlation analyses. Third, the drug sensitivity predictions are primarily based on *in vitro* data, requiring prospective clinical studies for validation of their translational value. Fourth, the causal relationship between epigenetic modifications and the immune microenvironment, as well as the molecular mechanisms underlying opposite prognostic implications in different cancer types, remains to be fully elucidated.

To address these limitations, future research should focus on: (1) integrating spatial transcriptomics data to better understand the spatial heterogeneity of epigenetic modifications while expanding sample sizes through multi-center collaboration; (2) conducting prospective clinical cohort studies with standardized data collection to validate the model’s predictive performance and therapeutic guidance value; (3) investigating the regulatory mechanisms of epigenetic modifications on the immune microenvironment through *in vitro* functional experiments and animal models; (4) exploring the formation mechanisms of tissue-specific epigenetic regulatory networks; and (5) establishing standardized protocols for model implementation and validation in clinical settings. We are actively addressing several of these limitations through an ongoing comprehensive clinical cohort study at our center, which will provide detailed clinical parameters and treatment outcomes for model validation. These continued efforts will contribute to further optimization of the prognostic prediction model and provide theoretical foundations for developing novel therapeutic strategies.

## 6 Conclusion

By integrating single-cell sequencing data with epigenetic regulatory networks, this study systematically revealed the molecular characteristics of epigenetic heterogeneity in OS and successfully established a Random Survival Forest-based prognostic prediction model. Our key findings include: (1) identification of five major cell types in the OS microenvironment, with significant epigenetic heterogeneity, particularly high epigenetic activity in OS cells and osteoclasts; (2) demonstration through pseudotime analysis that epigenetic modification levels significantly influence cell fate determination, with high- and low-score groups showing distinct spatial separation in evolutionary trajectories, indicating the crucial role of epigenetic regulation in tumor progression; (3) validation of the RSF model’s excellent predictive performance across multiple independent cohorts (internal validation AUC>0.997, external validation AUC = 0.832–0.929), with broad application potential across 19 different cancer types, particularly showing significant prognostic value in ACC, GBMLGG, and STAD.

Furthermore, our study systematically revealed the close association between epigenetic scores and the immune microenvironment, discovering significantly elevated stromal and immune scores in the low-risk group, suggesting that epigenetic modifications may influence disease prognosis through regulation of the tumor immune microenvironment. Additionally, drug sensitivity analysis identified seven compounds with potential therapeutic value, providing new options for risk stratification-based personalized treatment. These findings not only deepen our understanding of OS development mechanisms but also provide new theoretical foundations for clinical therapeutic decision-making, demonstrating significant translational medical value.

## Data Availability

The original contributions presented in the study are included in the article/Supplementary Material, further inquiries can be directed to the corresponding authors.

## References

[B1] Abdelgalil A. A. Alkahtani H. M. Al-Jenoobi F. I. (2019). Sorafenib. Profiles Drug Subst. Excip. Relat. Methodol. 44, 239–266. 10.1016/bs.podrm.2018.11.003 31029219

[B2] Aran V. Devalle S. Meohas W. Heringer M. Cunha Caruso A. Pinheiro Aguiar D. (2021). Osteosarcoma, chondrosarcoma and Ewing sarcoma: clinical aspects, biomarker discovery and liquid biopsy. Crit. Rev. Oncology/Hematology 162, 103340. 10.1016/j.critrevonc.2021.103340 33894338

[B3] Azevedo J. W. V. de Fernandes T. A. A. de M. José Veríssimo Fernandes J. de Azevedo J. C. V. Lanza D. C. F. Bezerra C. M. (2019). Biology and pathogenesis of human osteosarcoma. Oncol. Lett. 19 (2), 1099–1116. 10.3892/ol.2019.11229 31966039 PMC6955653

[B4] Becht E. McInnes L. Healy J. Dutertre C. A. Kwok I. W. H. Ng L. G. (2018). Dimensionality reduction for visualizing single-cell data using UMAP. Nat. Biotechnol. 37, 38–44. 10.1038/nbt.4314 30531897

[B5] Bejarano L. Jordāo M. J. C. Joyce J. A. (2021). Therapeutic targeting of the tumor microenvironment. Cancer Discov. 11 (4), 933–959. 10.1158/2159-8290.CD-20-1808 33811125

[B6] Budhavarapu V. N. Chavez M. Tyler J. K. (2013). How is epigenetic information maintained through DNA replication? Epigenetics Chromatin 6 (1), 32. 10.1186/1756-8935-6-32 24225278 PMC3852060

[B7] Chen C. Xie L. Ren T. Huang Y. Xu J. Guo W. (2021). Immunotherapy for osteosarcoma: fundamental mechanism, rationale, and recent breakthroughs. Cancer Lett. 500, 1–10. 10.1016/j.canlet.2020.12.024 33359211

[B8] Cheung L. C. Pan Q. Hyun N. Katki H. A. (2019). Prioritized concordance index for hierarchical survival outcomes. Stat. Med. 38 (15), 2868–2882. 10.1002/sim.8157 30957257 PMC6800570

[B9] Cole S. Gianferante D. M. Zhu B. Mirabello L. (2022). Osteosarcoma: a surveillance, epidemiology, and end results program-based analysis from 1975 to 2017. Cancer 128 (11), 2107–2118. 10.1002/cncr.34163 35226758 PMC11647566

[B10] Czarnecka A. M. Synoradzki K. Firlej W. Bartnik E. Sobczuk P. Fiedorowicz M. (2020). Molecular Biology of osteosarcoma. Cancers 12 (8), 2130. 10.3390/cancers12082130 32751922 PMC7463657

[B11] Dhanasekaran R. Deutzmann A. Mahauad-Fernandez W. D. Hansen A. S. Gouw A. M. Felsher D. W. (2022). The MYC oncogene - the grand orchestrator of cancer growth and immune evasion. Nat. Rev. Clin. Oncol. 19 (1), 23–36. 10.1038/s41571-021-00549-2 34508258 PMC9083341

[B12] Ettaieb M. Kerkhofs T. van Engeland M. Haak H. (2020). Past, present and future of epigenetics in adrenocortical carcinoma. Cancers (Basel) 12 (5), 1218. 10.3390/cancers12051218 32414074 PMC7281315

[B13] Grün D. van Oudenaarden A. (2015). Design and analysis of single-cell sequencing experiments. Cell 163 (4), 799–810. 10.1016/j.cell.2015.10.039 26544934

[B14] Hakobyan M. Binder H. Arakelyan A. (2024). Pan-cancer analysis of telomere maintenance mechanisms. J. Biol. Chem. 300 (6), 107392. 10.1016/j.jbc.2024.107392 38763334 PMC11225560

[B15] Hanahan D. (2022). Hallmarks of cancer: new dimensions. Cancer Discov. 12 (1), 31–46. 10.1158/2159-8290.CD-21-1059 35022204

[B16] Hong C. Yang S. Wang Q. Zhang S. Wu W. Chen J. (2021). Epigenetic age acceleration of stomach adenocarcinoma associated with tumor stemness features, immunoactivation, and favorable prognosis. Front. Genet. 12, 563051. 10.3389/fgene.2021.563051 33815458 PMC8012546

[B17] Hou W. Ji Z. Chen Z. Wherry E. J. Hicks S. C. Ji H. (2023). A statistical framework for differential pseudotime analysis with multiple single-cell RNA-seq samples. Nat. Commun. 14 (1), 7286. 10.1038/s41467-023-42841-y 37949861 PMC10638410

[B18] Huang Q. Liu Y. Du Y. Garmire L. X. (2021). Evaluation of cell type annotation R packages on single-cell RNA-seq data. Genomics Proteomics Bioinforma. 19 (2), 267–281. 10.1016/j.gpb.2020.07.004 PMC8602772 33359678

[B19] Jin S. Plikus M. V. Nie Q. (2024). CellChat for systematic analysis of cell-cell communication from single-cell transcriptomics. Nat. Protoc. 16, 180–219. 10.1038/s41596-024-01045-4 39289562

[B20] Kim L. C. Cook R. S. Chen J. (2017). mTORC1 and mTORC2 in cancer and the tumor microenvironment. Oncogene 36 (16), 2191–2201. 10.1038/onc.2016.363 27748764 PMC5393956

[B21] Korsunsky I. Millard N. Fan J. Slowikowski K. Zhang F. Wei K. (2019). Fast, sensitive and accurate integration of single-cell data with Harmony. Nat. Methods 16 (12), 1289–1296. 10.1038/s41592-019-0619-0 31740819 PMC6884693

[B22] Langfelder P. Horvath S. (2008). WGCNA: an R package for weighted correlation network analysis. BMC Bioinforma. 9, 559. 10.1186/1471-2105-9-559 PMC2631488 19114008

[B23] Li F. Zhang C. Zhang G. (2019). m6A RNA methylation controls proliferation of human glioma cells by influencing cell apoptosis. Cytogenet Genome Res. 159 (3), 119–125. 10.1159/000499062 31639789

[B24] Liang Y. Wang L. Ma P. Ju D. Zhao M. Shi Y. (2023). Enhancing anti-tumor immune responses through combination therapies: epigenetic drugs and immune checkpoint inhibitors. Front. Immunol. 14, 1308264. 10.3389/fimmu.2023.1308264 38077327 PMC10704038

[B25] Liberzon A. Birger C. Thorvaldsdóttir H. Ghandi M. Mesirov J. P. Tamayo P. (2015). The Molecular Signatures Database (MSigDB) hallmark gene set collection. Cell Syst. 1 (6), 417–425. 10.1016/j.cels.2015.12.004 26771021 PMC4707969

[B26] Liu W. Li L. Ye H. Tu W. (2017). Weighted gene co-expression network analysis in biomedicine research. Sheng Wu Gong Cheng Xue Bao 33 (11), 1791–1801. 10.13345/j.cjb.170006 29202516

[B27] Luo J. Xie Y. Zheng Y. Wang C. Qi F. Hu J. (2020). Comprehensive insights on pivotal prognostic signature involved in clear cell renal cell carcinoma microenvironment using the ESTIMATE algorithm. Cancer Med. 9 (12), 4310–4323. 10.1002/cam4.2983 32311223 PMC7300420

[B28] Manning B. D. Toker A. (2017). AKT/PKB signaling: navigating the network. Cell 169 (3), 381–405. 10.1016/j.cell.2017.04.001 28431241 PMC5546324

[B29] Medvedeva Y. A. Lennartsson A. Ehsani R. Kulakovskiy I. V. Vorontsov I. E. Panahandeh P. (2015). EpiFactors: a comprehensive database of human epigenetic factors and complexes. Database (Oxford) 2015, bav067. 10.1093/database/bav067 26153137 PMC4494013

[B30] Mialou V. Philip T. Kalifa C. Perol D. Gentet J. C. Marec-Berard P. (2005). Metastatic osteosarcoma at diagnosis: prognostic factors and long-term outcome--the French pediatric experience. Cancer 104 (5), 1100–1109. 10.1002/cncr.21263 16015627

[B31] Mirabello L. Troisi R. J. Savage S. A. (2009). International osteosarcoma incidence patterns in children and adolescents, middle ages and elderly persons. Int. J. cancer J. Int. du cancer. 125 (1), 229–234. 10.1002/ijc.24320 PMC3048853 19330840

[B32] Nie Z. Peng H. (2018). Osteosarcoma in patients below 25 years of age: an observational study of incidence, metastasis, treatment and outcomes. Oncol. Lett. 16 (5), 6502–6514. 10.3892/ol.2018.9453 30405789 PMC6202522

[B33] Recillas-Targa F. (2022). Cancer epigenetics: an overview. Arch. Med. Res. 53 (8), 732–740. 10.1016/j.arcmed.2022.11.003 36411173

[B34] Rigatti S. J. (2017). Random forest. J. Insur Med. 47 (1), 31–39. 10.17849/insm-47-01-31-39.1 28836909

[B35] Rodríguez P. C. Zea A. H. Ochoa A. C. (2003). Mechanisms of tumor evasion from the immune response. Cancer Chemother. Biol. Response Modif. 21, 351–364. 10.1016/s0921-4410(03)21018-8 15338755

[B36] Sebaugh J. L. (2011). Guidelines for accurate EC50/IC50 estimation. Pharm. Stat. 10 (2), 128–134. 10.1002/pst.426 22328315

[B37] Shaikh A. B. Li F. Li M. He B. He X. Chen G. (2016). Present advances and future perspectives of molecular targeted therapy for osteosarcoma. Int. J. Mol. Sci. 17 (4), 506. 10.3390/ijms17040506 27058531 PMC4848962

[B38] Shuai M. He D. Chen X. (2021). Optimizing weighted gene co-expression network analysis with a multi-threaded calculation of the topological overlap matrix. Stat. Appl. Genet. Mol. Biol. 20 (4-6), 145–153. 10.1515/sagmb-2021-0025 34757703

[B39] Siegel R. L. Giaquinto A. N. Jemal A. (2024). Cancer statistics, 2024. CA A Cancer J. Clin. 74 (1), 12–49. 10.3322/caac.21820 38230766

[B40] Song D. Yang D. Powell C. A. Wang X. (2019). Cell-cell communication: old mystery and new opportunity. Cell Biol. Toxicol. 35 (2), 89–93. 10.1007/s10565-019-09470-y 30815784

[B41] Sun L. Zhang H. Gao P. (2022). Metabolic reprogramming and epigenetic modifications on the path to cancer. Protein Cell 13 (12), 877–919. 10.1007/s13238-021-00846-7 34050894 PMC9243210

[B42] Talaei S. Mellatyar H. Asadi A. Akbarzadeh A. Sheervalilou R. Zarghami N. (2019). Spotlight on 17-AAG as an Hsp90 inhibitor for molecular targeted cancer treatment. Chem. Biol. Drug Des. 93 (5), 760–786. 10.1111/cbdd.13486 30697932

[B43] Tang N. Song W. X. Luo J. Haydon R. C. He T. C. (2008). Osteosarcoma development and stem cell differentiation. Clin. Orthop. Relat. Res. 466 (9), 2114–2130. 10.1007/s11999-008-0335-z 18563507 PMC2492997

[B44] Thanindratarn P. Dean D. C. Nelson S. D. Hornicek F. J. Duan Z. (2019). Advances in immune checkpoint inhibitors for bone sarcoma therapy. J. Bone Oncol. 15, 100221. 10.1016/j.jbo.2019.100221 30775238 PMC6365405

[B45] Tran H. T. N. Ang K. S. Chevrier M. Zhang X. Lee N. Y. S. Goh M. (2020). A benchmark of batch-effect correction methods for single-cell RNA sequencing data. Genome Biol. 21 (1), 12. 10.1186/s13059-019-1850-9 31948481 PMC6964114

[B46] Ullah R. Yin Q. Snell A. H. Wan L. (2022). RAF-MEK-ERK pathway in cancer evolution and treatment. Semin. Cancer Biol. 85, 123–154. 10.1016/j.semcancer.2021.05.010 33992782

[B47] Wang P. H. Liu C. H. Yang S. T. (2023). Risk-stratification system for preoperative evaluation. J. Chin. Med. Assoc. 86 (3), 259–261. 10.1097/JCMA.0000000000000860 36528800 PMC12755662

[B48] Yang J. Xu J. Wang W. Zhang B. Yu X. Shi S. (2023). Epigenetic regulation in the tumor microenvironment: molecular mechanisms and therapeutic targets. Signal Transduct. Target Ther. 8 (1), 210. 10.1038/s41392-023-01480-x 37217462 PMC10203321

[B49] Yang W. Soares J. Greninger P. Edelman E. J. Lightfoot H. Forbes S. (2013). Genomics of Drug Sensitivity in Cancer (GDSC): a resource for therapeutic biomarker discovery in cancer cells. Nucleic Acids Res. 41 (Database issue), D955–D961. 10.1093/nar/gks1111 23180760 PMC3531057

[B50] Yu S. Yao X. (2024). Advances on immunotherapy for osteosarcoma. Mol. Cancer 23, 192. 10.1186/s12943-024-02105-9 39245737 PMC11382402

[B51] Zeng D. Ye Z. Shen R. Yu G. Wu J. Xiong Y. (2021). IOBR: multi-omics immuno-oncology biological research to decode tumor microenvironment and signatures. Front. Immunol. 12, 687975. 10.3389/fimmu.2021.687975 34276676 PMC8283787

[B52] Zhou Y. Yang D. Yang Q. Lv X. Huang W. Zhou Z. (2021). Author Correction: single-cell RNA landscape of intratumoral heterogeneity and immunosuppressive microenvironment in advanced osteosarcoma. Nat. Commun. 12 (1), 2567. 10.1038/s41467-021-23119-7 33931654 PMC8087801

