# Supplementary figures and images for: Development and pan-cancer validation of an epigenetics-based random survival forest model for prognosis prediction and drug response in OS
Source: Front Pharmacol. 2025 Jan 22;16:1529525. doi: 10.3389/fphar.2025.1529525 (PMC11803151; doi:10.3389/fphar.2025.1529525)

**UMI Distribution by Sample**

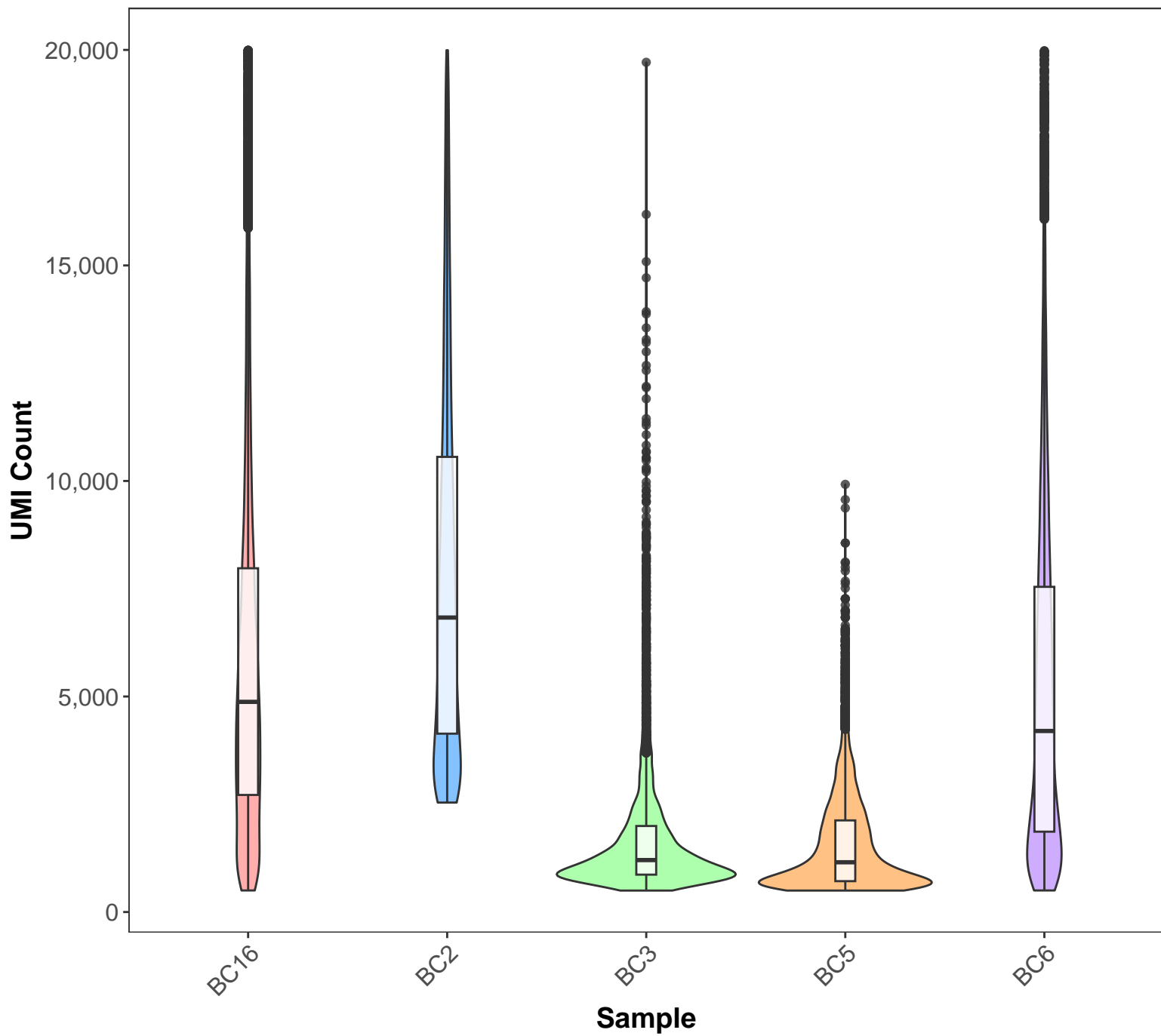

Supplement: Supplementary file 1 [file Image2.pdf]

**nFeature\_RNA**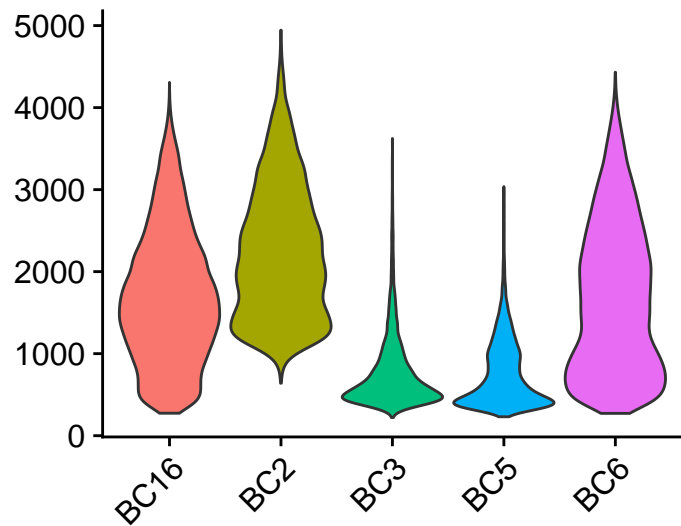**nCount\_RNA**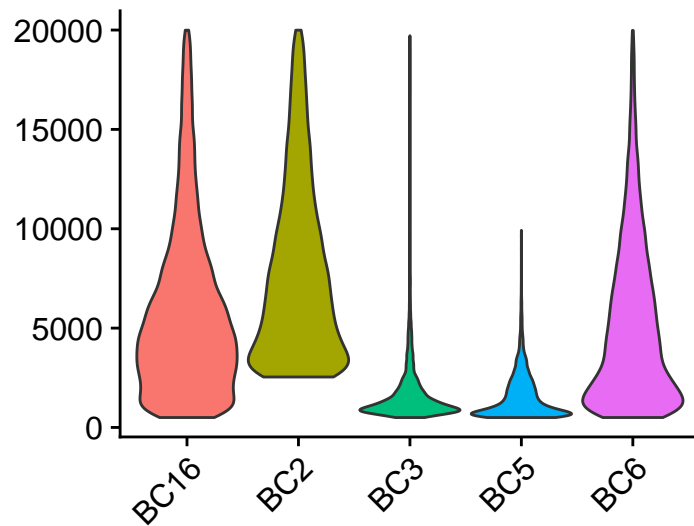**percent.mt**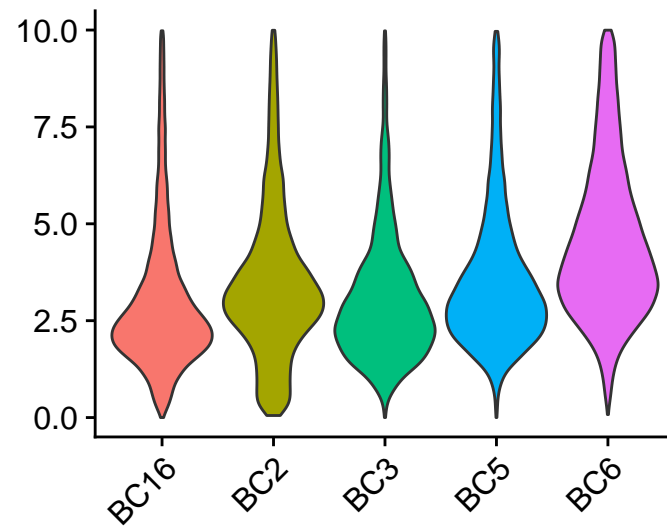**percent.rb**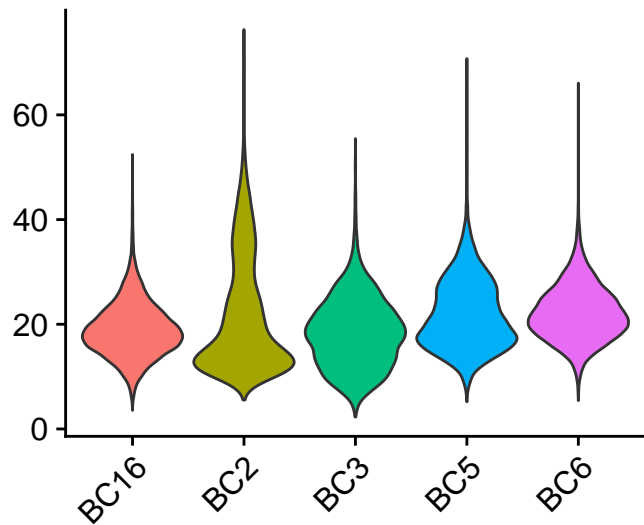**percent.HB**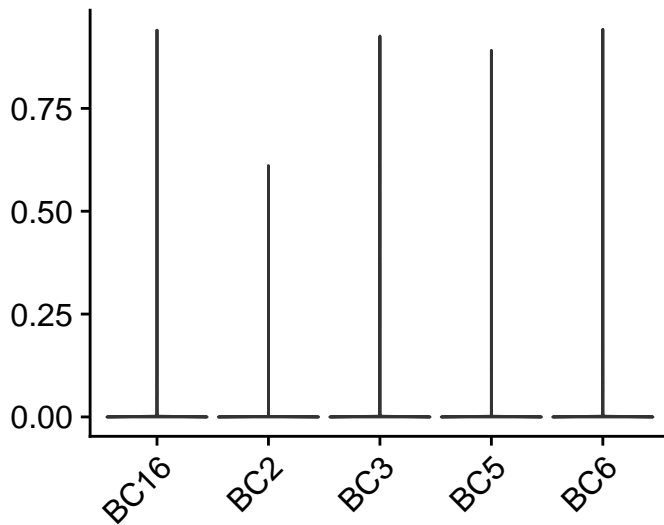

Supplement: Supplementary file 2 [file Image1.pdf]
